# Asymptomatic Congenital Hyperinsulinism due to a Glucokinase-Activating Mutation, Treated as Adrenal Insufficiency for Twelve Years

**DOI:** 10.1155/2017/4709262

**Published:** 2017-01-09

**Authors:** Kae Morishita, Chika Kyo, Takako Yonemoto, Rieko Kosugi, Tatsuo Ogawa, Tatsuhide Inoue

**Affiliations:** Center for Diabetes, Endocrinology and Metabolism, Shizuoka General Hospital, No. 4-27-1, Kita-Ando, Aoi-ku, Shizuoka, Shizuoka 420-8527, Japan

## Abstract

Congenital hyperinsulinism (CHI) caused by a glucokinase- (GCK-) activating mutation shows autosomal dominant inheritance, and its severity ranges from mild to severe. A 43-year-old female with asymptomatic hypoglycemia (47 mg/dL) was diagnosed as partial adrenal insufficiency and the administration of hydrocortisone (10 mg/day) was initiated. Twelve years later, her 8-month-old grandchild was diagnosed with CHI. Heterozygosity of exon 6 c.590T>C (p.M197T) was identified in a gene analysis of GCK, which was also detected in her son and herself. The identification of GCK-activating mutations in hyperinsulinemic hypoglycemia patients may be useful for a deeper understanding of the pathophysiology involved and preventing unnecessary glucocorticoid therapy.

## 1. Introduction

Congenital hyperinsulinism (CHI) is a condition that leads to recurrent hypoglycemia due to the inappropriate secretion of insulin by pancreatic islet *β* cells. Recent developments in gene analyses of CHI-related hypoglycemia have provided detailed information on each mutation detected [1]. Mutations are most frequently detected in the ABCC8 and KCNJ11 genes, which code for the two KATP-channel subunits SUR1 and Kir6.2, respectively [2, 3]. Mutations in the glucokinase (GCK), glutamate dehydrogenase (GLUD1), insulin receptor (INSR), hepatocyte nuclear factor 4a (HNF4A), and monocarboxylate transporter 1 (SLC16A1) genes have less commonly been reported to cause CHI and similar syndromes featuring hyperinsulinemic hypoglycemia [4–8].

Glucokinase (GCK), an enzyme that facilitates the phosphorylation of glucose to glucose-6-phosphate, is an important regulator of glucose homeostasis. GCK has been detected in the pancreas, liver, gut, and brain. In each of these organs, GCK plays an important role in the regulation of carbohydrate metabolism by acting as a glucose sensor in pancreatic *β* cells and promoting the synthesis of glycogen and triglycerides within the liver. A mutation in GCK leads to an inappropriate threshold for glucose-stimulated insulin secretion. Inactivating mutations have been shown to cause maturity-onset diabetes of the young (MODY), whereas activating mutations cause CHI-related hypoglycemia. The activation of glucokinase mutations increases the affinity of glucokinase for glucose and resets the threshold for glucose-stimulated insulin secretion. Thus, insulin continues to be produced at lower blood glucose levels. There appears to be little correlation between specific genotypes and the phenotype of GCK-activating mutations; although some case reports have claimed a relationship between severity of the hyperinsulinism and higher relative activity index of the expressed mutant enzyme, the glucose “set point” for most cases appears to be quite stable in a plasma glucose range of 55–65 mg/dL [9]. Information on the prevalence, natural history, and endocrinological effects of chronic hypoglycemia from GCK mutations is currently limited.

Here we describe the clinical course of GCK-activating mutation, especially focusing on the endocrinological aspects of chronic hypoglycemia. Identification of this mutation may be essential for preventing erroneous diagnosis, avoiding unnecessary glucocorticoid therapy, and having deeper understanding of the pathophysiology of hyperinsulinemic hypoglycemia.

## 2. Case Reports

The patient was a 56-year-old female. When she was 44 years old, she was referred to our hospital due to asymptomatic hypoglycemia with a plasma glucose level of 47 mg/dL, which was detected in a periodic health examination. Her glucose level at hospitalization was approximately 60 mg/dL and HbA1c level was 4.1% (Tables 1 and 2(a)). Neither reactive hypoglycemia nor hyperinsulinemia was detected by the oral glucose tolerance test (OGTT) (Table 2(b)). In the 18-hour fasting test, plasma glucose and immunoreactive insulin were 55 mg/dL and 3.0 *μ*IU/mL, respectively, suggesting that insulinoma was unlikely. The insulin tolerance test revealed the suppression of C-peptide as well as the poor responses of ACTH and cortisol (Table 2(c)). Although a circadian variation was maintained in plasma cortisol, her cortisol level in the early morning was slightly low (6.9 *μ*g/dL) (Table 2(d)). The response of cortisol in the rapid ACTH (250 *μ*g) stimulation test was at the lower limit of normal (cortisol peaked at 18.4 ≥ 18 *μ*g/dL) [10], while its response in the CRH stimulation test was relatively poor (cortisol peaked at 12.2 *μ*g/dL) (Tables 2(e) and 2(f)). The excretion of 17-hydroxycorticosteroids (17-OHCS) and 17-keto steroids (17-KS) in urine was 2.4 mg/day and 2.5 mg/day, respectively, both of which were below the normal ranges (2.6–7.8 mg/day and 3.1–8.8 mg/day, resp.). No morphological abnormalities were detected in the adrenal glands, liver, or pancreas on enhanced dynamic CT images. She was diagnosed with hypoglycemia due to partial adrenal insufficiency, and so the administration of hydrocortisone (10 mg/day) was initiated. Steroid supplementation slightly increased her fasting plasma glucose levels from 52 mg/dL to 65 mg/dL and HbA1c levels from 4.1% to 4.6%. These levels then plateaued for 12 years (Table 1). Further examinations were not conducted at that time.

Twelve years later, her 8-month-old grandchild was found to be hypoglycemic (46 mg/dL) while being treated for gastroenteritis. The grandchild's father, the son of our patient, was also diagnosed with asymptomatic hypoglycemia (below 60 mg/dL) in a health examination. A family history of asymptomatic hypoglycemia in three generations made the attending doctor of the grandchild suspect GCK-activating mutations. The DNA of her grandchild was analyzed by direct sequencing of the entire coding region and exon-intron boundaries of GCK in Osaka City General Hospital. The heterozygosity of exon 6 c.590T>C (p.M197T), a novel GCK mutation which has already been reported to be an activating mutation in vitro study [11], was identified in her grandson and her son.

CHI due to the same gene mutation was suspected in our patient, and she was hospitalized for a detailed examination when she was 56 years old. Reassessment of glucose diurnal rhythm revealed that her plasma glucose level was 59–83 mg/dL during the intake of 10 mg of hydrocortisone (Table 3(a)). When our patient decreased and discontinued oral hydrocortisone, her plasma glucose level was definitely lower (48 mg/dL) but no symptoms were observed (Table 3(a)). The circadian variation in cortisol was maintained without any intake of hydrocortisone (Table 3(b)). Although her basal cortisol level was relatively low (4.5–8.8 *μ*g/dL), cortisol responses to the ACTH and CRH stimulation tests were within the normal ranges; peaks in serum cortisol were observed at 24.5 *μ*g/dL and 15.8 *μ*g/dL, respectively (Tables 3(c) and 3(d)).

**Figure 1:**
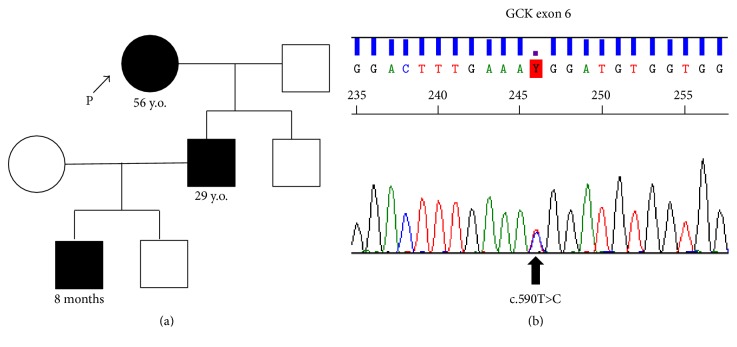
(a) Pedigree tree of the patient. (b) GCK gene analysis of the patient (grandmother). The heterozygosity of exon 6 c.590T>C (p.M197T), a novel GCK-activating mutation, was identified.

After obtaining written informed consent, a GCK gene analysis was conducted. The sequencing of exon 6 in her DNA revealed the same mutation as her son and grandchild, and she was diagnosed with congenital hyperinsulinemic hypoglycemia (Figure 1). Since there seems to be no correlation between genotype and phenotype of GCK mutation, we were unable to estimate the severity of hypoglycemia according to the mutation site [9, 12]. However, the severity of hypoglycemia was classified as mild based on her physical and mental states at the age of 43 in the absence of any treatment. Therefore, we decided to stop prescribing hydrocortisone. A favorable course has been followed for several years after the discontinuation of oral hydrocortisone.

## 3. Discussion

We encountered a family with CHI due to a novel mutation in GCK, with an 8-month-old infant as the index case. This GCK mutation itself has already been reported previously as a case report of the grandson [13]. We demonstrated here endocrinological aspects before and after steroid treatment in the patient with GCK-activating mutation, suggesting when and how we should suspect the GCK mutation as differential diagnoses of hypoglycemia.

Glucokinase-activating mutations result in hyperinsulinemic hypoglycemia. The diagnosis and treatment of hyperinsulinemic hypoglycemia have markedly advanced in recent years [14]. Severe hypoglycemia in infancy causes neurological dysfunctions and requires appropriate management. On the other hand, some cases of congenital hyperinsulinism are mild and overlooked until adulthood [15]. CHI related to a GCK-activating mutation was suspected in this case due to the presence of autosomal dominant inheritance and hyperinsulinism, with her blood glucose level being relatively low without symptoms. Difficulties are often associated with diagnosing CHI because, as in this case, some cases not accompanied by hyperinsulinemic hypoglycemia even though GCK gene mutations are present [12].

Glucokinase is a key regulatory enzyme in pancreatic *β*-cells. It phosphorylates glucose as a second substrate to form glucose-6-phosphate (G6P) as a first step in the glycolytic pathway. Since it plays a crucial role in the regulation of insulin secretion and is termed the glucose sensor in pancreatic *β*-cells, it is understandable that a mutation in GCK leads to an inappropriate threshold for glucose-stimulated insulin secretion. A glucokinase-activating mutation is one of the rare variants of congenital hyperinsulinism (CHI), and only 12 activating GCK mutations have been described and identified in 8 families and 7 individuals to date [12]. GCK gene mutations have been associated with various grades of symptoms such that many mild cases may be left undiagnosed until adulthood [16]. No criteria currently exist for the treatment of asymptomatic CHI.

A gene analysis involving GCK needs to be conducted when hypoglycemia with an unknown etiology is encountered. Although a family history is very important, there may be cases of new unnoticed-familial mutations. Information on genetic mutations, except for GCK, generally assists in predicting prognoses and responses to CHI treatments. In contrast, since the GCK mutation in the same family had a variety of phenotypes, we were unable to estimate the severity of hypoglycemia based on the mutation site [12]. It should be noted that our patient did not have any difficulties with stressful events, such as the delivery of her son, without any treatment. Therefore, the lower set point of hypoglycemia in our patient was not expected to become a serious problem throughout the rest of her life.

Our patient had been taking hydrocortisone for 12 years based on her poor response to the CRH stimulation test. According to previous findings on patients not only with diabetes [17, 18], but also without diabetes [19], hypoglycemia attenuated sympathoadrenal responses to declining plasma glucose concentrations, leading to hypoglycemia-associated autonomic failure (HAAF). Chronic hypoglycemia due to a GCK mutation may influence cortisol responses. Recent studies suggested that glucokinase in the hypothalamus and hindbrain participated in the activation of the norepinephrine and epinephrine neurons needed for the counterregulatory response [20, 21]. We speculated that not only HAAF but also a glucose sensing abnormality due to the activation of glucokinase in neuronal cells accounted for the counterregulatory hormone levels (basal and responses) of our patient.

An overdose of steroids is associated with a risk of side effects, including weight gain, hypertension, hyperlipidemia, and, sometimes, suppression of the HPA axis. Actually, her body weight increased by 3.8 kg (BMI: from 23.2 to 24.8) and blood pressure elevated (112/72 mmHg to 149/88 mmHg) during the 12-year treatment (Table 1). Two months after discontinuation of steroids, she had lost 1.8 kg in weight and her blood pressure decreased to 125/80 mmHg. Though 10 mg of oral hydrocortisone is within the physiological range, her circadian variation of cortisol must have been unnatural [22]. Considering these facts, 10 mg of oral hydrocortisone probably bears some responsibility with weight gain and hypertension.

In conclusion, the identification of GCK-activating mutations in hyperinsulinemic hypoglycemia patients may be useful for a deeper understanding of the pathophysiology involved and preventing unnecessary glucocorticoid therapy. More precise interpretations are needed while treating hypoglycemia and adrenal dysfunction.

## Figures and Tables

**Table 1:** Data of the patient.

	First visit	12 years later
Age (years)	44	56
Body weight (kg)	51	54.8
Height (cm)	148	148
BMI	23.2	24.8
Blood pressure (mmHg)	112/72	149/88
Fasting plasma glucose	52 mg/dL (2.8 mmol/L)	65 mg/dL (3.6 mmol/L)
Fasting serum insulin (*μ*IU/mL)	2.0	7.5
HbA1c (%)	4.1	4.6
Total cholesterol (mg/dL)	179	224
Cortisol (*μ*g/dL) (at 8:00 AM)	6.9–7.9	4.5–8.8
ACTH (pg/mL) (at 8:00 AM)	23.8–25.2	15.0–21.3

**Table:** (a) Glucose diurnal rhythm.

Clock time	7:30	10:00	11:30	14:00	17:30	20:00	23:00
Plasma glucose							
(mg/dL)	57	59	62	64	61	84	64
(mmol/L)	3.1	3.2	3.4	3.5	3.3	4.6	3.5

**Table:** (b) OGTT (oral glucose tolerance test) performed with 75 g glucose.

Time (min)	0	30	60	90	120
Plasma glucose					
(mg/dL)	62	119	120	84	75
(mmol/L)	3.4	6.6	6.6	4.6	4.1
Insulin (*μ*U/mL)	2.0	24.3	45.5	38	17.9

**Table:** (c) Plasma glucose, c-peptide, glucagon, cortisol, and ACTH responses to insulin tolerance test (0.05 U/kg BW of Humulin R).

Time (min)	0	15	30	45	60	75	90	120
Plasma glucose								
(mg/dL)	53	36	30	48	55	53	52	53
(mmol/L)	2.9	2.0	1.6	2.6	3.0	2.9	2.8	2.9
C-peptide (ng/mL)	1.2	0.7	0.5	0.5	0.5	0.4	0.4	0.5
Glucagon (pg/mL)	213	230	297	232	207	174		174
ACTH (pg/mL)	40	32.8	41.3	42.4	29.5	24.4	17.2	20.1
Cortisol (*μ*g/dL)	6.6	13.2	12	15	12.8	11.9	11.2	8.4

Normal values.

Glucagon 70–174 (pg/mL), ACTH: 7.2–63.3 (pg/mL), and cortisol: 8.0–18.0 (*μ*g/dL).

Interpretation of results.

The normal peak ACTH value poststimulation should be an increment no less than 50 pg/mL at 60′. Baseline cortisol values <5 *µ*g/dL are diagnostic of adrenal insufficiency. The normal peak cortisol value poststimulation should be an increment no less than 7 *µ*g/dL and a maximal level >20 *µ*g/dL at 30′.

**Table:** (d) Circadian variation in cortisol.

Clock time	8:00	17:00	23:00
ACTH (pg/mL)	23.8	14.3	14.5
Cortisol (*μ*g/dL)	6.9	2.0	>1.0

**Table:** (e) Cortisol response to the rapid ACTH stimulation test (250 *μ*g, intravenous bolus).

Time (min)	0	30	60
Cortisol (*μ*g/dL)	7.9	16.5	18.4

Interpretation of results.

The normal peak cortisol value poststimulation should be an increment no less than 18 *µ*g/dL.

**Table:** (f) Cortisol and ACTH responses to the CRH stimulation test (100 *μ*g, intravenous bolus).

Time (min)	0	15	30	60	90	120
ACTH (pg/mL)	35.2	49.3	54.6	54	45.9	37.8
Cortisol (*μ*g/dL)	10.4	11.4	12.2	12.2	11.6	11.4

Interpretation of results.

The normal peak ACTH value poststimulation should be an increment no less than 20 pg/mL. Cortisol should be an increment no less than 5 *µ*g/dL.

**Table:** (a) Glucose diurnal rhythm.

Clock time	7:30	11:30	17:30	20:00	23:00
*Plasma glucose *					
10 mg hydrocortisone (8:00)					
(mg/dL)	63	80	59	83	81
(mmol/L)	3.5	4.4	3.2	4.6	4.5
5 mg hydrocortisone (8:00)					
(mg/dL)	58	68	67	84	80
(mmol/L)	3.2	3.7	3.7	4.6	4.4
no hydrocortisone					
(mg/dL)	48	64	57	77	67
(mmol/L)	2.6	3.5	3.1	4.2	3.7

**Table:** (b) Circadian variation in cortisol.

Clock Time	8:00	17:00	23:00
ACTH (pg/mL)	21.3	10.2	8.3
Cortisol (*μ*g/dL)	8.8	2.8	1.1

**Table:** (c) Cortisol response to the ACTH rapid stimulation test (250 *μ*g, intravenous bolus).

Time (min)	0	30	60
Cortisol (*μ*g/dL)	6.8	22.2	24.5

Interpretation of results.

The normal peak cortisol value poststimulation should be an increment no less than 18 *µ*g/dL.

**Table:** (d) Cortisol and ACTH responses to the CRH stimulation test (100 *μ*g, intravenous bolus).

Time (min)	0	15	30	60	90	120
ACTH (pg/mL)	15	62.8	75.3	53.6	38	33.8
Cortisol (*μ*g/dL)	4.5	9	15.8	15.5	13	12.6

Interpretation of results.

The normal peak ACTH value poststimulation should be an increment no less than 20 pg/mL. Cortisol should be an increment no less than 5 *µ*g/dL.
